# Supplementary material for: The hexosamine biosynthetic pathway rescues lysosomal dysfunction in Parkinson’s disease patient iPSC derived midbrain neurons
Source: Nat Commun. 2024 Jun 19;15:5206. doi: 10.1038/s41467-024-49256-3 (PMC11186828; doi:10.1038/s41467-024-49256-3)
Supplement: Supplementary file 1 — Supplementary Information [file 41467_2024_49256_MOESM1_ESM.pdf]

Supplementary Information
Supplementary Figures and Figure Legends

Figure S1

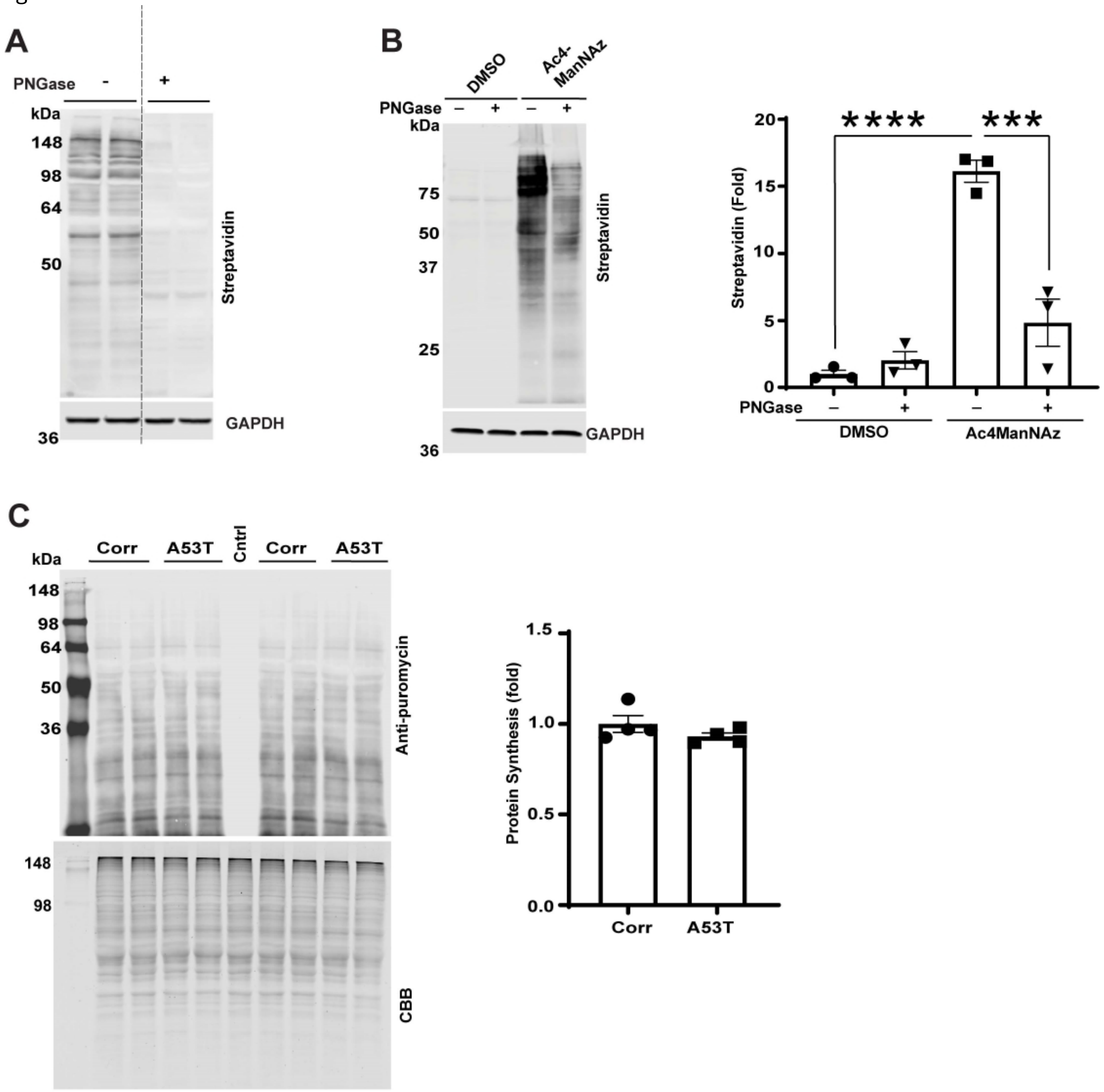

Figure S1. Con-A specifically detects N-glycosylated Proteins and ManNAz is incorporated into N-glycosylated proteins. A) H4 cell lysates were subjected to PNGase-F digestion and run on a 10% SDS-PAGE along with control lysate and subjected to western blotting against biotinylated Con-A, followed by detection with IRDye labeled streptavidin antibody. GAPDH is used as a loading control. B) Metabolic labeling of Ac4ManNAz differentiated SHSY5Y cells (Retinoic acid 10 μM for 5 days), followed by cell lysis at 96 hours (h). Labeled glycans were conjugated to biotin using biotin-phosphine. Lysates were subjected to PNGase digestion; undigested and digested lysates were subjected to western blot analysis. Biotinylated proteins were detected by IRDye labeled streptavidin antibody. GAPDH is a loading control (n=3). C) Protein synthesis remains unaltered in A53T iPSn. Western blot image shows newly synthesized proteins labeled with puromycin using the SUnSET

technique. Corr and A53T iPSn were treated with puromycin (5 µg/mL) for 1 h. Quantifications are shown on right (n=4). Lysate from corrected neurons not treated with puromycin was used as a negative control for puromycin incorporation. Scatter plots represent measurements from individual tissue culture wells. For all quantifications, values are the mean +/- SEM, *p<0.05, **p<0.01, ***p<0.001, ****p<0.0001. ANOVA-Tukey test was used for panel B, and Student's two-sided t-test was used panel C.

Figure S2.

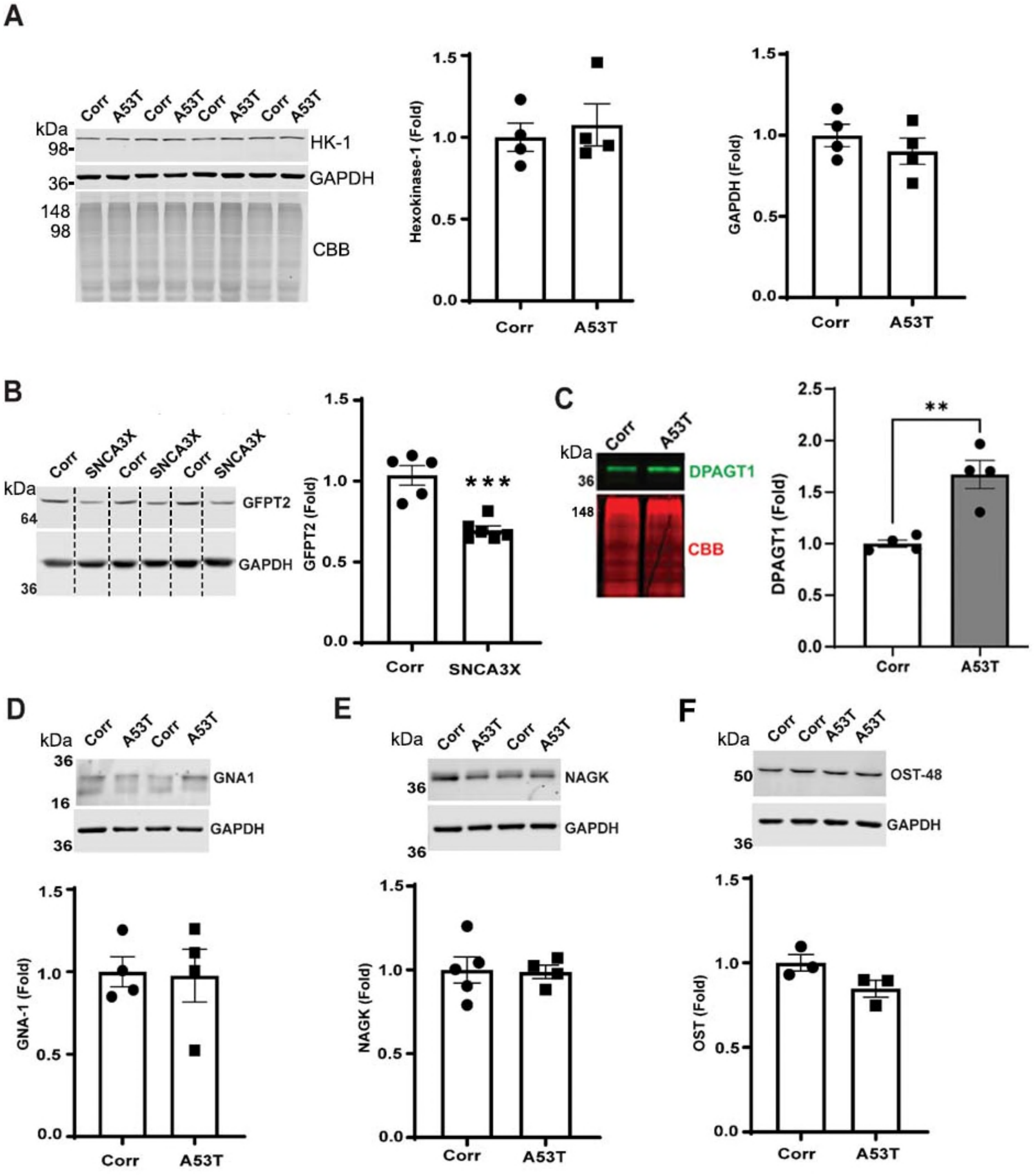

Figure S2. Quantification of enzymes involved in glycolysis or the HBP. A) Western blot analysis of HK-1 in iPSn (Corr and A53T at d90), using GAPDH loading controls. Quantification is shown on the right (n=4). GAPDH levels are also quantified and normalized to CBB, which remain unchanged between Corr and A53T iPSn. B) Western blot analysis of GFPT2 in iPSn (*SNCA*-3x (line 3X-2) and Corr) with GAPDH as a loading control. Quantifications are shown on the right (Corr n=5,*SNCA-3X*=6). C-F) Western blot analysis of DPAGT1 (n=4), GNA1 (n=4), NAGK (n=4) and OST (n=3) in iPSn (Corr and A53T at d90), using GAPDH as a loading control. Quantification is shown on the right. Scatter plots represent measurements from individual tissue culture wells. For all

quantifications, values are the mean +/- SEM, *p<0.05, **p<0.01, ***p<0.001, ****p<0.0001. Student's two-sided t-test was used for all comparisons *p<0.05.

Figure S3

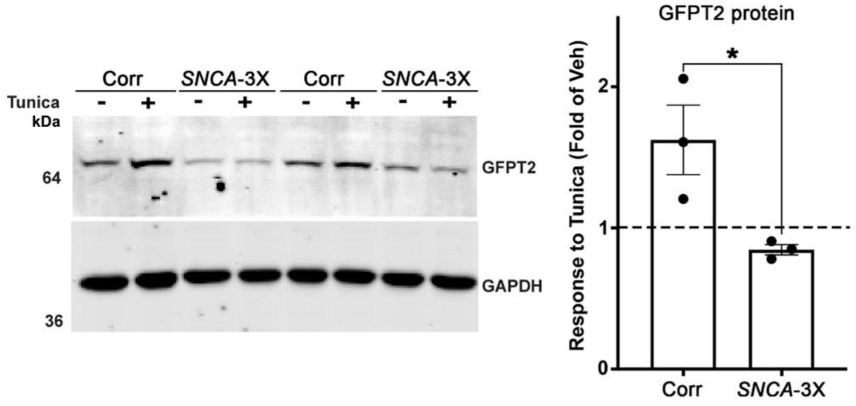

Figure S3. GFPT2 response to Tunicamycin-induced upregulation is compromised in *SNCA*-3x iPSn. Western blot analysis of GFPT2 protein in PD iPSn and matching isogenic control lines (*SNCA* 3X-1 and Corr) at day 90 after treatment with vehicle (DMSO) and tunicamycin (5 ug/ml) for 24 h. GAPDH was used as a loading control. Quantifications are shown on the right (n=3). Scatter plots represent measurements from individual tissue culture wells. For all quantifications, values are the mean +/- SEM, *p<0.05. Student's two-sided t-test.

Figure S4

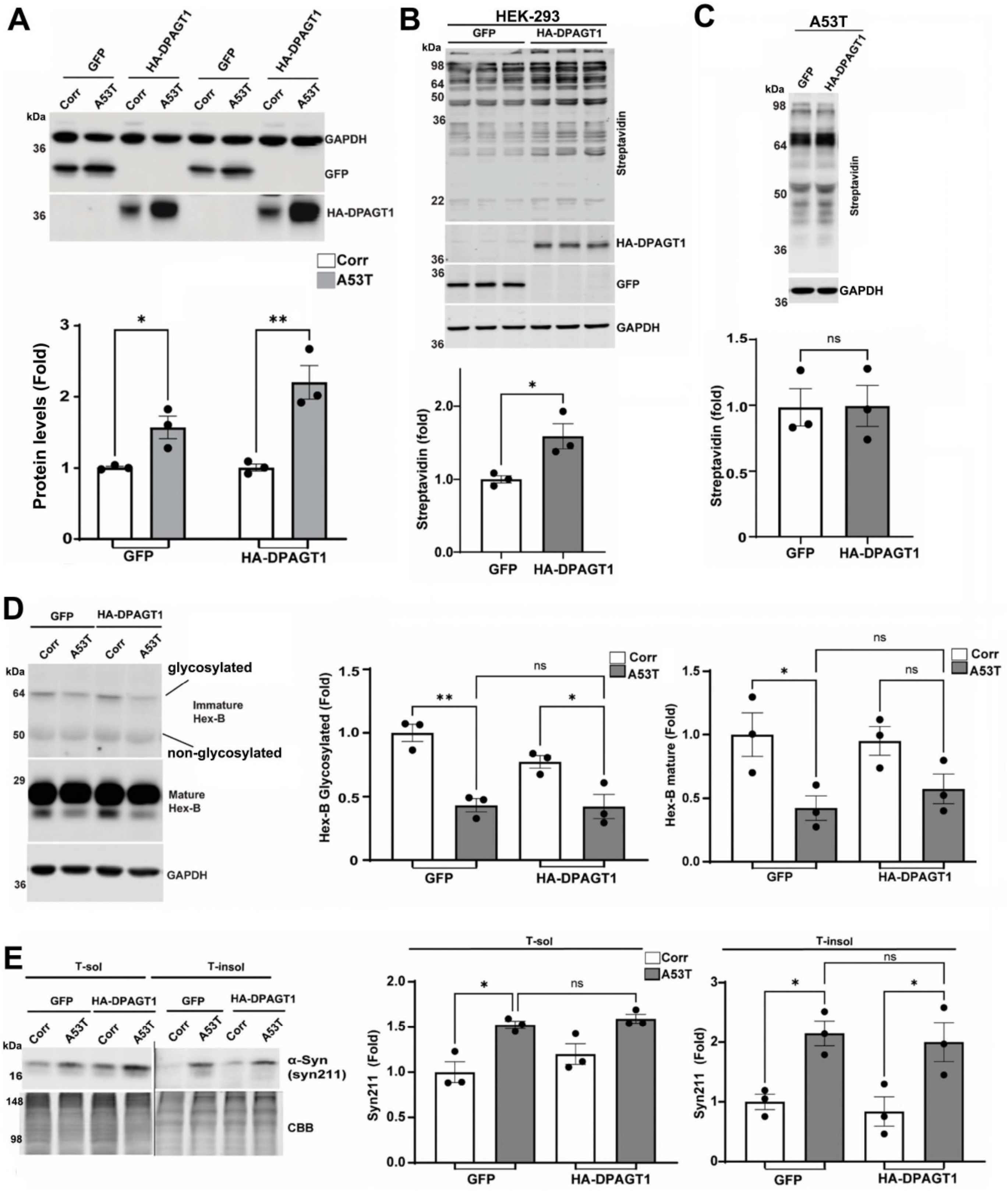

Figure S4. Overexpression of DPAGT1 does not improve N-glycosylation or lysosomal phenotypes in A53T iPSn. A) A53T or isogenic corrected controls (Corr) were transduced to overexpress HA-tagged DPAGT1 by lentiviral infection (MOI 3) at day 90 and analyzed by western blot for HA or DPAGT1 at day 105 (15 dpi) (n=3). B) HEK-293 cells were transiently transfected with the lenti-constructs followed by quantification of N-glycosylation (using Con-A Biotin / streptavidin) to confirm that functional enzyme is expressed (n=3). C) A53T iPSn were transduced

with lenti-HA-DPAGT1 as in panel A, followed by quantification of N-glycosylated proteins as described in panel B. D) Western blot analysis of HA-DPAGT1 transduced Corr or A53T iPSn to quantify maturation of Hex B. Quantification is on the right (n=3). E) Sequential extraction / western blot analysis of a-syn in Triton X-100 Soluble (T-sol) or insoluble (T-insol) fractions using syn211 antibody. Quantification is on the right (n=3). Scatter plots represent measurements from individual culture wells. For all quantifications, values are the mean +/- SEM, *p<0.05, **p<0.01, ***p<0.001, ****p<0.0001. Student's two-sided t-test was used for panels A, B and C. ANOVA with Tukey's test was used for panels D, and E.

Figure S5.

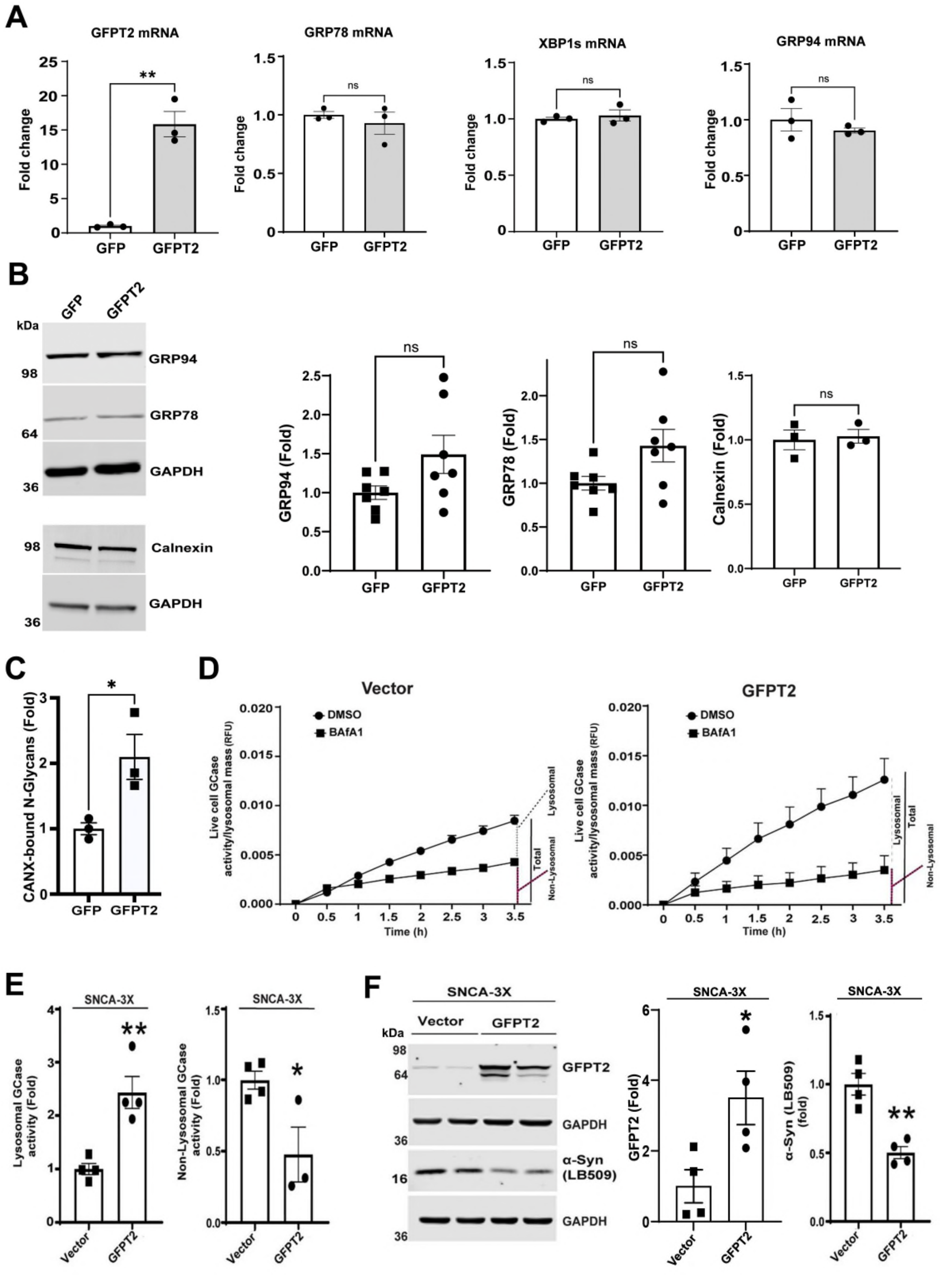

A
GFPT2 mRNA
GRP78 mRNA
XBP1s mRNA
GRP94 mRNA
Fold change
**
ns
GFP
GFPT2
B
kDa
GRP94
GRP78
GAPDH
Calnexin
GRP94 (Fold)
GRP78 (Fold)
Calnexin (Fold)
C
CANX-bound N-Glycans (Fold)
*
D
Vector
GFPT2
DMSO
BAfA1
Live cell GCase activity/lysosomal mass (RFU)
Time (h)
Lysosomal
Total
Non-Lysosomal
E
SNCA-3X
Lysosomal GCase activity (Fold)
Non-Lysosomal GCase activity (Fold)
Vector
F
α-Syn (LB509)
GFPT2 (Fold)
α-Syn (LB509) (fold)

Figure S5. GFPT2 overexpression in PD iPSn does not directly upregulate ER chaperones or XBP1s, but enhances lysosomal GCase activity and reduces pathological a-Syn. A) Q-RT-PCR analysis of mRNA levels in A53T iPSn transduced to overexpressed GFPT2 using the same conditions described in Figure 5 (n=3). B) Western blot analysis of ER chaperones in A53T iPSn transduced to overexpressed GFPT2 using the same conditions described in Figure 5 (n=3 for calnexin; n=7 for GRP94 and GRP78). GRP94 and GRP78 were analyzed on the same blot, while Calnexin was analyzed form a different blot. Each blot shows corresponding GAPDH levels as an indication of loading. C) Calnexin (CANX) binding activity was measured in PD iPSn (*SNCA*-3X) infected with GFP or GFPT2 as in panel A of Figure 5. Quantification of N-glycan binding was done by Con-A pull-down / CANX western blot (n=3 culture wells). D) Analysis of lysosomal and non-lysosomal GCase activity in live *SNCA*-3X iPSn infected with vector and GFPT2 lentivirus at MOI-3. Fluorescent GCase substrate degradation (PFB-FD-Gluc) was evaluated in a microplate reader for 3 h and normalized to lysosomal mass (dextran, cascade blue). Activity within acidic cellular compartments was determined by quantifying the response to bafilomycin A1 (Baf A1). Quantifications for the total GCase activity (circle plots of DMSO condition) and lysosomal GCase (the area between the DMSO and BafA1 curves). The area under BafA1 curve (square plots) represents the non-lysosomal GCase activity (n=3-4). RFU, relative fluorescent units. E) Lysosomal GCase was quantified by calculating the area between the DMSO and BafA1 curves and expressed as fold change compared to Vector infected cultures (n=3-4). F) Western blot analysis of GFPT2 and a-syn in *SNCA*-3x iPSn infected with lentivirus expressing vector and GFPT2 at MOI-3, analyzed at 15 days after infection. GAPDH is a loading control. a-Syn was detected with LB509 antibody and quantification is shown on the right (n=4). Scatter plots represent measurements from individual culture wells. For all quantifications, values are the mean +/- SEM, *p<0.05, **p<0.01, ns, not significant. Student's two-sided t-test.

Figure S6.

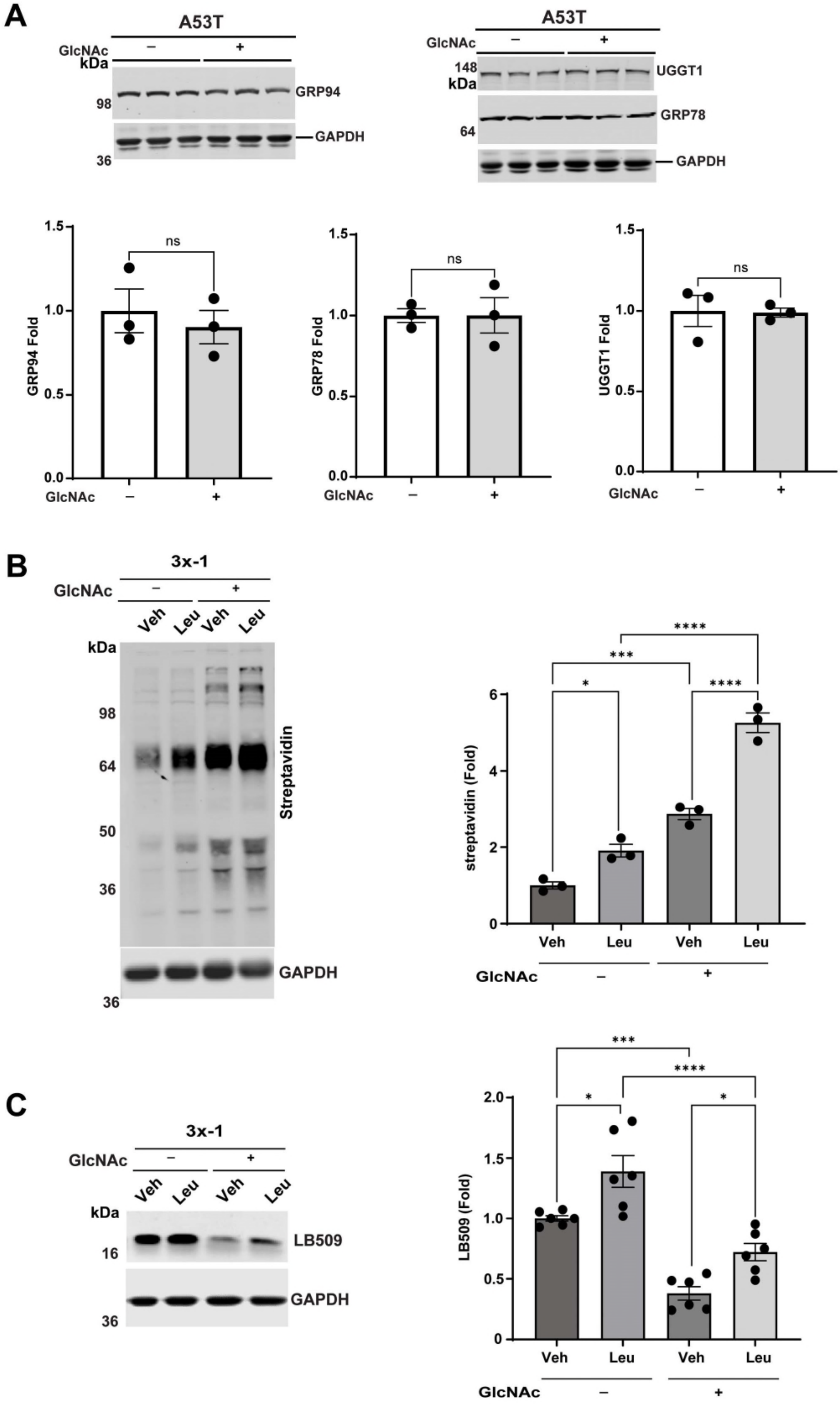
A
A53T
GlcNAc
kDa
98
36
GRP94
GAPDH
148
kDa
64
UGGT1
GRP78
GAPDH
GRP94 Fold
GRP78 Fold
UGGT1 Fold
ns
B
3x-1
Veh
Leu
98
64
50
36
Streptavidin
GAPDH
streptavidin (Fold)
*
***
****
C
3x-1
LB509
16
GAPDH
36
LB509 (Fold)

Figure S6. GlcNAc Supplementation does not alter ER chaperones or UGGT1 but elevates N-glycosylation and enhances lysosomal degradation of a-syn in PD iPSn. A) Western blot analysis of GlcNAc-treated A53T iPSn to quantify protein levels of GRP78, GRP94, and UGGT1 (n=3). B) Western blot of N-glycosylated proteins using biotinylated Con-A, in *SNCA*-3x iPSn (day 117) treated with 10 mM GlcNAc for 7 days followed by Leupeptin (100 μM) treatment for 24 h to inhibit lysosomal function. GAPDH is a loading control. Quantification is shown on the right (n=3). C) Western blot analysis of a-syn (antibody LB509) from Triton X-100 soluble (T-sol) fraction in *SNCA*-3x iPSn in the presence or absence of GlcNAc and Leu, using GAPDH as loading control. Quantification is shown on the right (n=6). Scatter plots represent measurements from individual culture wells. For all quantifications, values are the mean +/- SEM, *p<0.05, **p<0.01, ***p<0.001, ****p<0.0001. ANOVA with Tukey's post hoc test was used for panels B and C, or Students two-sided t-test in panel A.

Supplementary Table S1
Key Resources Table. This table includes a list of essential reagents used in this study.

REAGENT or RESOURCE	SOURCE	IDENTIFIER
Antibodies		
Rabbit polyclonal anti-alpha synuclein (C-20)	Santa Cruz	Cat #sc-7011-R RRID: AB_2192953
Concanavalin-A, biotinylated	Vector Laboratories	Cat #B-1005-5
Mouse monoclonal anti-alpha synuclein (LB509)	Abcam	Cat #ab27766 RRID: AB_727020
Rabbit polyclonal anti-GFP	Sigma Aldrich	Cat #G1544 RRID: AB_439690
Mouse monoclonal anti-alpha synuclein (303)	Biolegend	Cat #824301 RRID: AB_2564879
Mouse monoclonal anti-β3-tubulin	Biolegend	Cat #802001 RRID: AB_2564645
Rabbit polyclonal anti-GFPT2	Abcam	Cat # ab190966 RRID: AB_2868470
Mouse monoclonal anti-GAPDH	Millipore Sigma	Cat #CB1001 RRID: AB_2107426
Rabbit polyclonal anti-glucocerebrosidase (GCase)	Sigma	Cat #G4171 RRID: AB_1078958
Mouse Monoclonal anti-puromycin	Millipore sigma	Cat # MABE343 RRID: AB_2566826
Mouse monoclonal anti-Hexosaminidase B (HexB)	Santa Cruz	Cat #sc-376781 RRID: AB_2909474
Neurofilament	Biolegend	Cat #SMI-312R RRID: AB_2314906
Mouse monoclonal anti-oligosaccharyltransferase (OST48) antibody	Santa Cruz	Cat # sc-74408 RRID: AB_1125745
Mouse monoclonal anti- Hexokinase-1 (HXK I)	Santa Cruz	Cat #sc-46695
Mouse monoclonal anti – phosphoglucosamine acetylase (GNA1) antibody	Santa Cruz	Cat #sc- sc-374519 RRID: AB_10986418
Mouse monoclonal anti – GlcNAc kinase (NAGK) antibody	Santa Cruz	Cat #sc- 390499
Rabbit polyclonal anti-GRP78	Novus	Cat #NBP1-06274; RRID: AB_1555284
Rabbit anti-Calnexin antibody	Cell signaling	Cat #2433S; RRID: AB_2243887

Rabbit anti-DPAGT1 antibody	Thermo Fisher Scientific	Cat# PA5-72704, RRID: AB_2718558)
Mouse monoclonal anti- UGGT1 antibody	Santa Cruz	Cat# sc-374565, RRID: AB_11008073)
N-Azidoacetylmannosamine-tetraacylated (Ac4ManNAz)	Sigma	Cat # 900917
Secondary antibody: Alexa Fluor 680 Goat anti-mouse IgG secondary (H+L) For Western Blot	Invitrogen	Cat #A21058 RRID: AB_2535724
Secondary antibody: IRDye 800 goat anti-mouse IgG secondary (H+L) For Western Blot	Li-Cor Biosciences	Cat #926-32210 RRID: AB_621842
Secondary antibody: I IRDye 800 goat anti-rabbit IgG secondary (H+L) For Western Blot	Li-Cor Biosciences	Cat #926-32211 RRID: AB_621843
Secondary antibody: IRDye 800 CW anti-streptavidin, For Western Blot	Li-Cor Biosciences	Cat #92632230
Bacterial and virus strains		
lenti-pER4 (HIV, replication incompetent)	Mazzulli et al., 2011	N/A
pER4 GFPT2 lentivirus	This paper	N/A
pLenti-GIII-CMV- DPAGT1-HA (C-term)	This paper	N/A
pLV[Exp]-EGFP: T2A: Puro-EF1A>hXBP1[NM_001079539.1]	This paper	N/A
Biological samples		
Human brain tissue of control, DLB, DLB + AD patients	Northwestern University Alzheimer's disease pathology core (CNADC). Stojkovska *et al.*, 2022	N/A
Mouse Brain tissue from litter-matched controls and A53T mice	This paper, line originally described in Giasson et al., Neuron, 2002	N/A
Chemicals, peptides, and recombinant proteins		
Bafilomycin A1	Santa Cruz	Cat #SC-201550
Cascade Dextran Blue	Life Technologies	Cat #D1976
Conduritol β epoxide (CBE)	Millipore	Cat #234599
Doxycycline (DOX)	Sigma	Cat #D3447
Fetal bovine serum (FBS), heat-inactivated	Thermo Fisher Scientific	Cat #10438026
Geneticin (G418)	Thermo Fisher Scientific	Cat #10131027
L-glutamine	Gibco	Cat #25030081
Hygromycin B	Thermo Fisher Scientific	Cat #10687010
Paraformaldehyde (10%, methanol-free)	Polysciences, Inc.	Cat #40181
Penicillin / Streptomycin	Thermo Fisher Scientific	Cat #10378016
Phenylmethylsulfonyl fluoride (PMSF)	Sigma	Cat #78830
Protease Inhibitor Cocktail (PIC)	Roche	Cat #11836170001
N-Lauroylsarcosine sodium salt (sarkosyl)	Sigma	Cat #L9150
Sodium dodecyl sulfate (SDS)	Sigma	Cat #L4509
Sodium orthovanadate (Na_3VO_4)	Sigma	Cat #450243
Sodium fluoride (NaF)	Sigma	Cat #201154
Sucrose	Sigma	Cat #S1888
Triton X-100	Sigma	Cat #T8787
Tunicamycin (Tunic)	EMD Millipore / Calbiochem	Cat # 654380

5-(pentafluoro-benzoylamino) fluorescein di-ß-D-glucopyranoside (PFB-FDGluc)	Life Technologies	Cat #P11947
MKC8866	MCE Medchem Express	Cat#HY-104040
Critical commercial assays		
CellTag 700	Li-Cor Biosciences	Cat #926-41090
Endoglycosidase H	New England Biolabs	Cat #P0702L
HIV1-p24 Antigen ELISA Kit	Zeptometrix	Cat #0801111
DC™ Protein Assay Kit	Bio-Rad	#500
RevertAid First Strand cDNA Synthesis Kit	Thermo Fisher Scientific	Cat #K1621
Rneasy Mini Prep Kit	QIAGEN	Cat #74104
Quantitative RT-PCR: DPAGT1 (Hs00609752_m1)	Thermo Fisher Scientific	Cat #4331182
Quantitative RT-PCR: GRP78 (ID: Hs99999174_m1)	Thermo Fisher Scientific	Cat #4331182
Quantitative RT-PCR: GFPT2 (Hs01049570_m1)	Thermo Fisher Scientific	Cat #4331182
Quantitative RT-PCR: XBP1U(Hs02856596_m1)	Thermo Fisher Scientific	Cat #4331182
Quantitative RT-PCR: XBP1-S (ID: Hs03929085_g1)	Thermo Fisher Scientific	Cat #4331182
Quantitative RT-PCR: HSP90B1 / GRP94 (Hs00427665_g1)	Thermo Fisher Scientific	Cat #4331182
Quantitative RT-PCR: GBA1 (ID: Hs00164683_m1)	Thermo Fisher Scientific	Cat #4331182
Quantitative RT-PCR: HexB (Hs01077594_m1) and	Thermo Fisher Scientific	Cat #4331182
Quantitative RT-PCR: ACTB (Hs01060665_g1)	Thermo Fisher Scientific	Cat #4331182
QuikChange XL Site-Directed Mutagenesis Kit	Agilent	Cat #200517
Deposited data		
Experimental models: Cell lines		
H4 neuroglioma cells	Mazzulli et al., 2011; From: Pamela McLean (Mayo Clinic, Jacksonville, Florida, USA)	N/A
GM15010 (*SNCA* Triplication, 3x-1)	Stojkovska *et al.*, 2022; clinical and other information can be obtained from the Coriell Cell Repository	N/A
ND00196 (*SNCA* Triplication, 3x-2)	Stojkovska *et al.*, 2022; clinical and other information can be obtained from the Coriell Cell Repository	N/A
ND34391 (*SNCA* Triplication, Est. 3X).	Stojkovska *et al.*, 2022; Mazzulli et al., 2016a; Zunke et al., 2018; Cuddy et al., 2019; Coriell Cell Repository	N/A
A53T a-syn and isogenic control	Soldner et al., Cell, 2011	N/A
SH-SY5Y cells, female origin	Cuddy et al., 2019	ATCC Cat #CRL22-66

Experimental models: Organisms/strains		
Oligonucleotides- See Table S4		
Recombinant DNA		
pENTR223-GFPT2 (ccsbBroadEn_07515)	Addgene	Cat # HsCD000031302707 7
lenti-pER4	Mazzulli et al., 2011	N/A
pER4 GFPT2 lentivirus	This paper	N/A
pLV[Exp]-EGFP: T2A: Puro-EF1A>hXBP1[NM_001079539.1]	This paper	VB900007-1013mfa
pLenti-GIII-CMV-C-term-HA (Backbone for DPAGT1)		LV141214
Software and algorithms		
GraphPad Prism V6.0 software	GraphPad	https://www.graphpad.com/scientific-software/prism/
Odyssey software (Image Studio V3.1.4)	Li-Cor Biosciences	https://www.licor.com/bio/image-studio/
Snap gene V5.3 software	SnapGene	https://www.snapgene.com
Other		
Intercept blocking buffer	Li-Cor Biosciences	Cat #927-70001
Lenti-X concentrator	Clontech	Cat #631232
Matrigel	Fisher	Cat #CB-40234
mTeSR1 media	StemCell Technologies	Cat #85850
Neurobasal SM1 media	Thermo Fisher Scientific	Cat #21103-049
NeuroCult SM1 supplement	StemCell Technologies	Cat #05711
NeutrAvidin agarose beads	Thermo Fisher Scientific	Cat #29204
PVDF transfer membrane, 0.45 μm pore size	Millipore	Cat #IPFL00010
X-tremeGENE HP DNA Transfection Reagent	Roche	Cat #6366236001
Glucose Assay Kit- WST	Dojindo Molecular Technologies, INC	Cat # G264
