# Supplementary material for: The hexosamine biosynthetic pathway rescues lysosomal dysfunction in Parkinson’s disease patient iPSC derived midbrain neurons
Source: Nat Commun. 2024 Jun 19;15:5206. doi: 10.1038/s41467-024-49256-3 (PMC11186828; doi:10.1038/s41467-024-49256-3)
Supplement: Supplementary file 3 — Description of Additional Supplementary Files [file 41467_2024_49256_MOESM3_ESM.pdf]

Description of Additional Supplementary Files

File name: Supplementary Data 1
Description: Pathological and Clinical Information of clinically confirmed healthy controls, DLB, BLBD, PSP, and AD cases.
