# Supplementary material for: The hexosamine biosynthetic pathway rescues lysosomal dysfunction in Parkinson’s disease patient iPSC derived midbrain neurons
Source: Nat Commun. 2024 Jun 19;15:5206. doi: 10.1038/s41467-024-49256-3 (PMC11186828; doi:10.1038/s41467-024-49256-3)
Supplement: Supplementary file 5 — Reporting Summary [file 41467_2024_49256_MOESM5_ESM.pdf]

nature portfolio

Corresponding author(s): Mazzulli, Joseph R

Last updated by author(s): May 1, 2021

Reporting Summary

Nature Portfolio wishes to improve the reproducibility of the work that we publish. This form provides structure for consistency and transparency in reporting. For further information on Nature Portfolio policies, see our Editorial Policies and the Editorial Policy Checklist.

Statistics

For all statistical analyses, confirm that the following items are present in the figure legend, table legend, main text, or Methods section.

n/a	Confirmed	
[ ]	[x]	The exact sample size (*n*) for each experimental group/condition, given as a discrete number and unit of measurement
[ ]	[x]	A statement on whether measurements were taken from distinct samples or whether the same sample was measured repeatedly
[ ]	[x]	The statistical test(s) used AND whether they are one- or two-sided<br>*Only common tests should be described solely by name; describe more complex techniques in the Methods section.*
[x]	[ ]	A description of all covariates tested
[ ]	[x]	A description of any assumptions or corrections, such as tests of normality and adjustment for multiple comparisons
[ ]	[x]	A full description of the statistical parameters including central tendency (e.g. means) or other basic estimates (e.g. regression coefficient) AND variation (e.g. standard deviation) or associated estimates of uncertainty (e.g. confidence intervals)
[ ]	[x]	For null hypothesis testing, the test statistic (e.g. *F*, *t*, *r*) with confidence intervals, effect sizes, degrees of freedom and *P* value noted<br>*Give P values as exact values whenever suitable.*
[x]	[ ]	For Bayesian analysis, information on the choice of priors and Markov chain Monte Carlo settings
[x]	[ ]	For hierarchical and complex designs, identification of the appropriate level for tests and full reporting of outcomes
[x]	[ ]	Estimates of effect sizes (e.g. Cohen's *d*, Pearson's *r*), indicating how they were calculated

Our web collection on statistics for biologists contains articles on many of the points above.

Software and code

Policy information about availability of computer code

Data collection	Western blots were scanned using a Li-Cor Biosciences infrared imaging system or Azure Biosciences Sapphire scanner. Protein band intensities was quantified by the ImageStudio software ver 3.1.<br>Real-time PCR was performed on an Applied Biosystems 7500 Fast system using pre-designed Taqman-primer probe from Thermo Fisher Scientific.<br>For Live-cell lysosomal GCase activity assay was performed using Molecular Devices Spectramax M5 plate reader.
Data analysis	Graphing and statistics was performed using Prism 9 (GraphPad) software.

For manuscripts utilizing custom algorithms or software that are central to the research but not yet described in published literature, software must be made available to editors and reviewers. We strongly encourage code deposition in a community repository (e.g. GitHub). See the Nature Portfolio guidelines for submitting code & software for further information.

Data

Policy information about availability of data

All manuscripts must include a data availability statement. This statement should provide the following information, where applicable:

- Accession codes, unique identifiers, or web links for publicly available datasets
- A description of any restrictions on data availability
- For clinical datasets or third party data, please ensure that the statement adheres to our policy

All the data generated or analyzed supporting the conclusions of the study are included in the manuscript or its supporting data.

Human research participants

Policy information about studies involving human research participants and Sex and Gender in Research.

Reporting on sex and gender	N/A
Population characteristics	N/A
Recruitment	N/A
Ethics oversight	N/A

Note that full information on the approval of the study protocol must also be provided in the manuscript.

Field-specific reporting

Please select the one below that is the best fit for your research. If you are not sure, read the appropriate sections before making your selection.

[x] Life sciences [ ] Behavioural & social sciences [ ] Ecological, evolutionary & environmental sciences

For a reference copy of the document with all sections, see nature.com/documents/nr-reporting-summary-flat.pdf

Life sciences study design

All studies must disclose on these points even when the disclosure is negative.

Sample size	The sample sizes were based on previously published studies (Cuddy et al., 2019, Mazzulli et al, PNAS 2016) with at least three biological replicates per condition.
Data exclusions	For culture studies in some cases, obvious differences in culture behavior, morphology, or other easily identifiable features were used as exclusion criteria.
Replication	To address rigor and reproducibility for each model, all assays were performed from distinct iPSC passages using at least 3 biological replicates. Each batch of iPSC-neurons was subjected to strict quality control analysis to assure the integrity of the cultures, and accumulation of pathogenic alpha-synuclein. The most essential assays were performed in multiple PD lines to assess differences in multiple genetic backgrounds. All attempts to repeat the experiment were successful
Randomization	iPSC midbrain cultures were plated in random areas of the culture well and, when applicable, randomly assigned a treatment or control group
Blinding	studies were not blinded.

Reporting for specific materials, systems and methods

We require information from authors about some types of materials, experimental systems and methods used in many studies. Here, indicate whether each material, system or method listed is relevant to your study. If you are not sure if a list item applies to your research, read the appropriate section before selecting a response.

Materials & experimental systems

n/a	Involved in the study
☐	☒ Antibodies
☐	☒ Eukaryotic cell lines
☒	☐ Palaeontology and archaeology
☐	☒ Animals and other organisms
☐	☒ Clinical data
☒	☐ Dual use research of concern

Methods

n/a	Involved in the study
☒	☐ ChIP-seq
☒	☐ Flow cytometry
☒	☐ MRI-based neuroimaging

Antibodies

Antibodies used

Primary antibodies: Rabbit polyclonal anti-alpha synuclein (C-20) (Santa Cruz Cat #sc-7011-RRID: AB_2192953, 1:1000); Concanavalin-A, biotinylated (Vector Laboratories Cat #B-1005-5,1:1000); Mouse monoclonal anti-alpha synuclein (LB509) (Abcam Cat #ab27766 RRID: AB_727020,1:1000); Rabbit polyclonal anti-GFP (Sigma Aldrich Cat #Gl544 RRID: AB_439690,1:2000); Mouse monoclonal anti-alpha synuclein (303) (Biolegend Cat #824301 RRID: AB_2564879,1:1000); Mouse monoclonal anti-β3-tubulin (Biolegend Cat #802001 RRID: AB_2564645,1:10,000); Rabbit polyclonal anti-GFPT2 (Abeam Cat# ab190966 RRID: AB_2868470,1:500); Mouse monoclonal anti-GAPDH (Millipore Cat #CBl00l RRID: AB_2107426,1:10,000); Rabbit polyclonal anti-glucocerebrosidase (GCase) (Sigma Cat #G4171 RRID: AB_1078958,1:500); Mouse Monoclonal anti- puromycin (Millipore sigma Cat# MABE343 RRID: AB_2566826,1:1000); Mouse monoclonal anti-Hexosaminidase B (HexB) (Santa Cruz Cat #sc-376781 RRID: AB_2909474,1:1000); Neurofilament (Biolegend Cat #SMl-312R RRID: AB_2314906,1:1000); Mouse monoclonal anti- oligosaccharyltransferase (OST48) antibody (Santa Cruz Cat# sc-74408 RRID: AB_1125745,1:1000); Mouse monoclonal anti- Hexokinase-1 (HXKl) (Santa Cruz Cat #sc-46695,1:2000); Mouse monoclonal anti - phosphoglucosamine acetylase (GNAl) antibody (Santa Cruz Cat #sc- sc-374519 RRID: AB_10986418,1:500); Mouse monoclonal anti-GlcNAc kinase (NAGK) antibody (Santa Cruz Cat #sc- 390499,1:1000) Rabbit polyclonal anti-GRP78 (Novus Cat #NBPl-06274; RRID: AB_1555284,1:1000); Rabbit anti-Calnexin antibody (Cell signaling Cat #2433S; RRID: AB_2243887,1:500); Rabbit anti-DPAGT1 antibody (Thermo Fisher Scientific Cat# PA5-72704, RRID: AB_2718558,1:500); Mouse monoclonal anti- UGGT1 antibody (Santa Cruz Cat# sc-374565, RRID: AB_11008073,1:500).

Secondary antibodies: Secondary antibody: Alexa Fluor 680 Goat anti-mouse lgG secondary (H+L) (lnvitrogen Cat #A21058 RRID: AB_2535724,1:10000); lrdye 800CW goat anti-mouse lgG secondary (H+L) (Li-Car Biosciences Cat #926-32210 RRID: AB_621842,1:5000); lrdye 800CW goat anti-rabbit lgG secondary (H+L) (Li-Car Biosciences Cat #926-32211 RRID: AB_621843,1:10,000); IRDye800 CW anti-streptavidin (Li-Car Biosciences Cat #92632230,1:5000).

Validation

We have used commercially available antibodies that have been extensively tested for specificity. Validation of the commercial antibodies used in this study is available on manufacturer's websites. Most of these antibodies have been previously used and validated by multiple labs. Some antibodies were validated in the current study using overexpression of the encoding gene or using Knock out lysate in western blot in the case of α-synuclein (SNCA KO lysate).

Rabbit polyclonal anti-alpha synuclein (C-20) (Santa Cruz Cat #sc-7011-RRID: AB_2192953) and Mouse monoclonal anti-alpha synuclein (303) (Biolegend Cat #824301 RRID: AB_2564879) were Validated using α-synuclein KO lysate in Figure 6G; Concanavalin-A, biotinylated (Vector Laboratories Cat #B-1005-5), Validated using PNGase-F digestion Figure S1A; Mouse monoclonal anti-alpha synuclein (LB509) (Abcam Cat #ab27766 RRID: AB_727020 https://www.abcam.com/a1pha-synuclein- antibody-lb-509-ab27766.html); Rabbit polyclonal anti-GFP (Sigma Aldrich Cat #Gl544 RRID: AB_439690, Validated by GFP Overexpression in Figure 5A https://www.sigmaaldrich.com/US/en/product/sigma/g1544); Mouse monoclonal anti-β 3-tubulin (Biolegend Cat #802001 RRID: AB_2564645, https://www.biolegend.com/en-us/products/purified-anti-tubulin-beta-3-tubb3-anti body-115 79 GroupID=GROUP686); Rabbit polyclonal anti-GFPT2 (Abcam Cat# ab190966 RRID: AB_2868470, Validated by GFPT2 Overexpression Figure 5A); Mouse monoclonal anti-GAPDH (Millipore Cat #CBlO0l RRID: AB_2107426, https://www.emdmillipore.com/US/en/product/Anti-GAPDH-Mouse-mAb-6C5,EMD_BIO-CB1001); Rabbit polyclonal anti-glucocerebrosidase (GCase) (Sigma Cat #G4171 RRID: AB_1078958, https://www.sigmaaldrich.com/US/en/product/sigma/g4171); Mouse Monoclonal anti-puromycin (MilliporesigmaCat#MABE343RRID:AB_2566826,https://www.sigmaaldrich.com/US/en/product/mm/mabe343) Mouse monoclonal anti-Hexosaminidase B (Hex-B) (Santa Cruz Cat #sc-376781 RRID: AB_2909474, https://www.scbt.com/p/hexb-b-chain-antibody-d-9); Neurofilament (Biolegend Cat #SMl-312R RRID: AB_2314906, https://www.biolegend.com/en-us/products/purified-anti-neurofilament-marker-pan-axonal-cocktail-12811?GroupID=BLG15643); Mouse monoclonal anti-oligosaccharyltransferase (OST48) antibody (Santa Cruz Cat# sc-74408 RRID: AB_1125745, https://www.scbt.com/p/ost48-antibody-e-9); Mouse monoclonal anti-Hexokinase-1 (HXK I) (Santa Cruz Cat #sc-46695, https://www.scbt.com/p/hxk-i-antibody-g-1); Mouse monoclonal anti - phosphoglucosamine acetylase (GNAl) antibody (Santa Cruz Cat #sc-374519 RRID: AB_10986418, https://www.scbt.com/p/gnal-antibody-a-3); Mouse monoclonal anti - GlcNAc kinase (NAGK) antibody (Santa Cruz Cat #sc- 390499, https://www.scbt.com/p/glcnac-kinase-antibody-g-5) Rabbit polyclonal anti-GRP78 (Novus Cat #NBPl-06274; RRID: AB_1555284, https://www.novusbio.com products/grp 78-hspas-antibody_nbp1-06274); Rabbit anti-Calnexin antibody (Cell signaling Cat #2433S; RRID: AB_2243887, https://www.cellsignal.com/products/primary-antibodies/calnexin-antibody/2433); Rabbit anti-DPAGT1 antibody (Thermo Fisher Scientific Cat# PA5-72704, RRID: AB_2718558, https://www.thermofisher.com/antibody/product/DPAGT1-Antibody-Polyclonal/PA5-72704); Mouse monoclonal anti- UGGT1 antibody (Santa Cruz Cat# sc-374565, RRID: AB_11008073, https://www.scbt.com/p/uggt1-antibody-h-9).

Eukaryotic cell lines

Policy information about cell lines and Sex and Gender in Research

Cell line source(s)	H4 neuroglioma cells Mazzulli et al., 2011; From: Pamela McLean (Mayo Clinic, Jacksonville, Florida, USA) ;<br>GM15010 (SNCA Triplication, 3x-1) Stojkovska et al., 2022; clinical and other information can be obtained from the Coriell Cell Repository<br>ND00196 (SNCA Triplication, 3x-2) Stojkovska et al., 2022; clinical and other information can be obtained from the Coriell Cell Repository<br>ND34391 (SNCA Triplication, Est. 3X). Stojkovska et al., 2022; Mazzulli et al., 2016a; Zunke et al., 2018; Cuddy et al., 2019; Coriell Cell Repository<br>SNCA Triplication, line 1A Mazzulli et al, PNAS 2016<br>A53T alpha-synuclein and isogenic control gifted by Dr. R. Jaenisch (Whitehead Institute of MIT) Soldner et al., Cell, 2011<br>SH-SY5Y cells, female origin Cuddy et al., 2019 ATCC Cat #CRL22-66
Authentication	All iPSCs and cell lines have been previously described and characterized extensively. For authentication genotyping was performed for common disease-causing mutations published in (Mazzulli et al., 2011), pluripotency analysis, karyotype analysis, efficient differentiation into midbrain dopamine neurons, and absence of mycoplasma. H4 cells have been previously authenticated (Mazzulli et al., 2011) by analyzing expression of α-synuclein (α-syn) and absence of mycoplasma.
Mycoplasma contamination	All iPSC lines were routinely tested for mycoplasma contamination.
Commonly misidentified lines<br>(See ICLAC register)	None

Animals and other research organisms

Policy information about studies involving animals; ARRIVE guidelines recommended for reporting animal research, and Sex and Gender in Research

Laboratory animals	Our study was performed under the Northwestern University IACUC protocol number Number IS00021691. The line details are described in the methods section, Jackson stock number 004479, line M83 Vle/J on a B6;C3H background, using matched littermates as the controls.
Wild animals	N/A
Reporting on sex	We used samples from both male and female mice, although our studies used n=3 mice for each group and therefore we could not analyze statistical differences between males and females in this study. Our goal for this study was to define differences in GFPT2 levels based on the genotype alone. No sex differences in α-synuclein pathology or other phenotypes have been previously noted in this line
Field-collected samples	N/A
Ethics oversight	N/A

Note that full information on the approval of the study protocol must also be provided in the manuscript.

Clinical data

Policy information about clinical studies

All manuscripts should comply with the ICMJEguidelines for publication of clinical research and a completedCONSORT checklist must be included with all submissions.

Clinical trial registration	*Provide the trial registration number from ClinicalTrials.gov or an equivalent agency.*
Study protocol	*Note where the full trial protocol can be accessed OR if not available, explain why.*
Data collection	*Describe the settings and locales of data collection, noting the time periods of recruitment and data collection.*
Outcomes	*Describe how you pre-defined primary and secondary outcome measures and how you assessed these measures.*
